# Children’s screen use and school readiness at 4-6 years: prospective cohort study

**DOI:** 10.1186/s12889-022-12629-8

**Published:** 2022-02-23

**Authors:** Leigh M. Vanderloo, Magdalena Janus, Jessica A. Omand, Charles D.G. Keown-Stoneman, Cornelia M. Borkhoff, Eric Duku, Muhammad Mamdani, Gerald Lebovic, Patricia C. Parkin, Janis Randall Simpson, Mark S. Tremblay, Jonathon L. Maguire, Catherine S. Birken

**Affiliations:** 1 Child Health Evaluative Sciences, Hospital for Sick Children Research Institute, 686 Bay Street, ON M5G 0A4 Toronto, Canada; 2 Offord Centre for Child Studies, Department of Psychiatry and Behavioural Neurosciences, McMaster University, Hamilton, ON Canada; 3 Applied Health Research Centre, Li Ka Shing Knowledge Institute, St. Michael’s Hospital, Toronto, ON Canada; 4 Dalla Lana Faculty of Public Health, University of Toronto, Toronto, ON Canada; 5 Institute of Health Policy, Management and Evaluation, Dalla Lana School of Public Health, University of Toronto, Toronto, ON Canada; 6 Unity Health Toronto, Toronto, ON Canada; 7 Department of Medicine, Temetry Faculty of Medicine, University of Toronto, Toronto, ON Canada; 8 Leslie Dan Faculty of Pharmacy, University of Toronto, Toronto, ON Canada; 9 Division of Paediatric Medicine, Paediatric Outcomes Research Team (PORT), Hospital for Sick Children, Toronto, ON Canada; 10 Department of Family Relations and Applied Nutrition, University of Guelph, Guelph, ON Canada; 11 Healthy Active Living and Obesity Research, CHEO Research Institute, Ottawa, ON Canada; 12 Department of Pediatrics, St. Michael’s Hospital, Toronto, Ontario Canada

**Keywords:** School readiness, Early Development Instrument, Screen use, Language development, Cognitive development

## Abstract

**Background:**

The primary aim of this study was to determine if screen use in early childhood is associated with overall vulnerability in school readiness at ages 4 to 6 years, as measured by the Early Development Instrument (EDI). Secondary aims were to: (1) determine if screen use was associated with individual EDI domains scores, and (2) examine the association between screen use and EDI domains scores among a subgroup of high screen users.

**Methods:**

This prospective cohort study was carried out using data from young children participating in a large primary care practice-based research network in Canada. Logistic regression analyses were run to investigate the association between screen use and overall vulnerability in school readiness. Separate linear regression models examined the relationships between children’s daily screen use and each separate continuous EDI domain.

**Results:**

A total of 876 Canadian participants participated in this study. Adjusted logistic regression revealed an association between increased screen use and increased vulnerability in school readiness (*p* = 0.05). Results from adjusted linear regression demonstrated an association between higher screen use and reduced language and cognitive development domain scores (*p* = 0.004). Among high screen users, adjusted linear regression models revealed associations between increased screen use and reduced language and cognitive development (*p* = 0.004) and communication skills and general knowledge domain scores (*p* = 0.042).

**Conclusions:**

Screen use in early childhood is associated with increased vulnerability in developmental readiness for school, with increased risk for poorer language and cognitive development in kindergarten, especially among high users.

**Supplementary Information:**

The online version contains supplementary material available at 10.1186/s12889-022-12629-8.

## Introduction

The link between excessive sedentary behaviours and poor health outcomes across the lifespan is well-noted in the literature [1–3]. Early childhood is an important period in child development; high levels of daily sedentary behaviours may inimitably influence the health and learning outcomes during this period [4]. It is still unclear as to whether screen use should be touted as an aid or impediment during early childhood, and whether such decisions are dependent on the quantity or quality of screen use, or possibly both [5, 6]. A review (n = 76 studies) by Kostyrka-Allchorne et al. [7], which examined the association between television viewing, cognition and behaviour in children, reported that early onset of viewing and inappropriate content may be related to negative outcomes. A prospective longitudinal study by Pagani and colleagues [8] found that every additional hour of television exposure at 29 months corresponded to 7% and 6% unit decreases in classroom engagement and math achievement, respectively; and that exposure during this critical period may make a unique contribution to developmental risk. In contrast, review findings claim that educational TV may enhance learning, particularly for preschoolers [7]. Given the pervasiveness of screens among young children [9, 10], it is important to ascertain how screen time is associated with their development.

By school entry, researchers believe that close to 30% of Canadian children show some form of deficit or delay in developmental outcomes like language, socioemotional status, and communication [11, 12]. As concerns in early childhood development often worsen sans meaningful interference, the extent of children inadequately prepared for learning is worrisome [13] typically measured at school entry, school readiness centres around developmental areas related to children’s future success including physical, socioemotional, and language and cognitive factors [14].

Regardless the link between high screen use and negative physical and psychosocial outcomes in young children [15–18], mixed evidence concerning the use of screens for educational purposes may allude to some possible benefits of high-quality and interactive screen use [7, 19–22]. With the duration and availability of screen use increasing [23], as well as the context in which this screen-viewing takes places (intentional and unintentional exposure, background vs. foreground exposure, co-viewing, at home vs. school, etc.) [24, 25], many queries remain regarding their impact on children’s preparedness to begin their scholarly journey. Understandably, the link between screen use and development are complex and require further investigations; optimal child developmental trajectories and policy recommendations are dependent on the identification of modifiable behavioural factors in this young cohort.

Despite the growing pervasiveness of techno-dependence and screen use, there is limited evidence of its impact on school readiness or developmental health in young children. As such, the primary objective of this study was to determine whether screen use in early childhood was associated with overall vulnerability in school readiness at ages 4 to 6 years, as measured by the Early Development Instrument (EDI). Secondary objectives included determining whether screen use in early childhood was associated with scores in individual EDI domains as well as examining the association between screen use and scores in individual EDI domains in a subgroup of high screen users (exploratory).

## Methods

Using data from 2013 to 2020, a prospective cohort study was conducted with children 12 months to 6 years, who were enrolled in a large Canadian primary care practice-based research network – TARGet Kids! (http://www.targetkids.ca) [26]. Children were recruited at any age up to the age of 6 and were then assessed annually at a scheduled health visit with their family physician until adolescence. Consent was obtained for all participants from their parent/guardian prior to enrollment. Institutional approval from the appropriate research ethics boards (The Hospital for Sick Children, Unity Health, and respective participating school boards) was received for study materials and protocols. The present paper adheres to STROBE Guidelines for Cohort Studies [27], and all methods were carried out in accordance with relevant guidelines and regulations. Additional information regarding the cohort profile can be found elsewhere [28].

### Eligibility Criteria

This study included children whose parents/guardians had completed questionnaires on screen use and had the EDI completed during the second half of the school year by their teacher in kindergarten (junior kindergarten or senior kindergarten). Children without chronic health conditions except for asthma, severe developmental delays prior to enrollment, had a gestational age **≥ **32 weeks, and had English-speaking parents/guardians who provided consent to participate were eligible to participate in the study.

### Exposure Variable – Screen Use

The primary exposure of interest was children’s total daily screen use duration. These data were obtained from a standardized parent-reported questionnaire which was advised by the validated and long-standing Canadian Community Health Survey – a national cross-sectional survey that collects information related to health status, health care utilization and health determinants for the Canadian population [29]. Total daily screen use was derived by taking the sum of mean screen use (across all devices, e.g., TV, computer, video games, tablets, smartphones) during the week and weekend (i.e., average daily screen use on weekday*5 + average daily screen use on weekend day*2) / 7). Exposure data on screen use collected closest to the EDI outcome measure (but preceding it) were retained. Restricted cubic splines with 5 knots were used to test for non-linearity and to accommodate various shapes for the association of daily screen use in the models (*p*-value cut-off = 0.05).

### Outcome Variable – School Readiness

Used worldwide, the EDI is a teacher-completed evaluation of school readiness which has been validated for use with children in kindergarten [11, 30, 31]. Specific to the Canadian province of Ontario, the kindergarten sample ranges from 3 to 6 years as kindergarten includes children in “junior kindergarten” (JK; where children enter in September of the calendar year they turn 4 years old) and “senior kindergarten” (SK; which children enter the year they turn 5). The EDI’s psychometric properties have been evaluated in Canada and in other countries, with scores being predictive of academic achievement and social relationships [11, 32–35]. The primary outcome of this study was children’s overall vulnerability on the EDI (0 = not vulnerable, 1 = vulnerable). Vulnerability was defined as being below the 10th percentile cut-off of a normative distribution on at least one of the EDI domains. Percentile cut-offs were based on published cut offs for SK [32] and JK [36].

The secondary outcome focused on the continuous scores on each of the EDI domains (0 = low ability to 10 = high ability). Specifically, the EDI is comprised of five developmental domains: *emotional maturity* (ability to think before acting, ability to deal with feelings at the age-appropriate level, ability to demonstrate empathetic responses to other people), *communication skills and general knowledge* (skills to communicate needs/wants in socially appropriate ways, symbolic use of language, storytelling, age-appropriate knowledge about the world around), *physical health and well-being* (gross and fine motor skills, adequate energy for classroom activities, independence in daily living skills), *social competence* (knowledge of acceptable public behaviour, ability to control own behaviour, appropriate respect for adult authority, cooperation with others, ability to play/work with others), and *language and cognitive development* (age-appropriate reading and writing skills, age-appropriate numeracy skills, ability to recite back specific pieces of information from memory) [11].

### Confounding Variables

Identified *a priori* from the literature [5, 11, 37] and collected by a parent-reported child health questionnaire, the following confounding variables were included: child’s sex, age at outcome and physical activity level, as well as maternal ethnicity and education, annual household income, and follow-up time. Children with special needs were identified by teachers using special needs designation reports.

### Statistical Analyses

R version 3.5.0 (R Core Team, Boston, MA) was used for all data cleaning and analyses [38]. Descriptive statistics were performed on of all variables entered into the models. Given the absence of accepted definitions or thresholds that identify ‘high screen users’ in this age group, histograms were used to examine the distribution of the screen use variable (exposure) and to determine the top 10% of screen users (i.e., high users) as a suitable cut-off. As some EDI data were collected during the second half of 2020, a sensitivity analysis was run to assess whether results meaningfully differed using the data collected pre- versus during the COVID-19 pandemic using an interaction with the primary exposure (total screen time).

To address the primary objective, odds ratios, 95% confidence intervals, and *p*-values were estimated from logistic regression analyses between total daily screen use and overall vulnerability in school readiness while adjusting for confounders. As informed by past research [39–42], interactions for sex and age with total daily screen use were included in the model [43, 44].

For the secondary objective, separate linear regression models were used to examine the relationships between children’s total daily screen use and each separate continuous EDI domain, adjusting for confounders. For the models of the continuous domain scores, a combination of bootstrap resampling and imputation (500 repetitions) was used to estimate the 95% confidence intervals and *p*-values for each model [45]. Specifically, bootstrap resampling was performed to address the non-normality of the residuals, and within each bootstrap resample we performed a single imputation to address missing covariate data. Imputations were performed using the mice package in R [46], as missing data was assumed to be missing-at-random (MAR). Reported missingness for each variable was under 15% [47]. Age and sex were entered as interaction terms [39–42]. Linear regression models were also used to investigate the associations between high users and each of the five EDI domains.

## Results

**Fig. 1:**
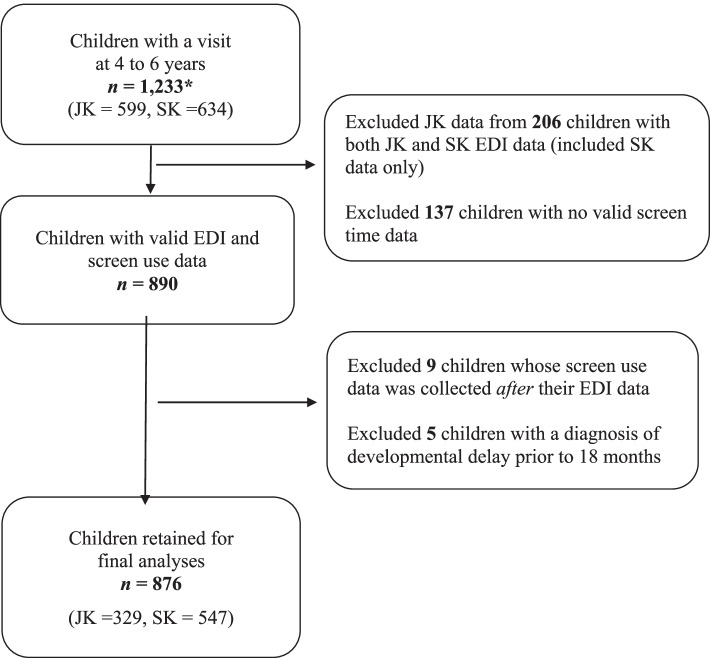
Study participant flow chart. JK = junior kindergarten; SK = senior kindergarten. *Children in JK and SK recruited between 2013 and 2020, whereby 139 cases were collected during COVID-19 pandemic (March-November 2020)

A total of 876 children (329 in JK and 547 in SK) with complete outcome data at follow-up were included in the final analyses of this study (Fig. 1). The average participant age at exposure and outcome was 3.7 (*SD* = 0.4) years and 5.4 (*SD* = 0.3) years, respectively, with the mean duration between exposure and outcome being 11.3 (*SD* = 2.1) months. Approximately 52.8% of participants were male and 69.2% had mothers who self-reported European ethnicity (Table 1). Most parents who completed the survey had a college or university degree (91.6%) and 60.0% reported a household income below $150,000. Mean daily screen use was 4.8 (2.3) hours. The top 10% of daily screen users (*n* = 85), reported 8+ hours of daily screen use. A total of 131 participants (17%) were classified as “vulnerable” on the EDI tool.

**Table 1:** Characteristics of participating children (*n* = 876)

Variable	Total Sample (*n* = 876)	Children not vulnerable on the EDI (*n* = 745)	Children vulnerable on the EDI (*n* = 131)
Age at exposure (years), mean (*SD)*	3.7 (0.4)	3.9 (0.6)	3.4 (0.2)
Age at outcome (years), mean (*SD)*	5.4 (0.3)	5.8 (0.2)	5.6 (0.5)
Sex, Male, *n*(%)	463 (52.8)	362 (48.6)	101 (77.1)
Birth weight (kilograms), mean (*SD*)	3.3 (0.7)	3.4 (0.5)	2.9 (0.8)
Ethnicity, *n*(%)			
*European Ancestry*	606 (69.2)	570 (76.4)	36 (27.7)
*South Asian and South-East Asian*	27 (3.1)	20 (2.7)	7 (5.3)
*East Asian*	32 (3.7)	22 (3.0)	10 (7.6)
*Other*	37 (4.2)	28 (3.8)	9 (11.8)
*Mixed Ethnicity*	174 (19.9)	102 (13.7)	72 (55.4)
Maternal Education, *n*(%)			
*College/ University*	802 (91.6)	705 (98.5)	37 (35.2)
*High School or less*	74 (8.4)	11 (1.5)	68 (64.8)
Household Income, *n*(%)			
*$0 to $56,999*	129 (14.7)	83 (11.3)	41 (44.1)
*$60,000 to $99,999*	156 (17.8)	122 (16.6)	23 (24.7)
*$100,000 to $149,999*	241 (27.5)	217 (29.5)	10 (10.8)
*$150,000 +*	350 (40.0)	313 (42.6)	19 (14.0)
Total daily screen use (hours), mean *(SD)*	4.8 (2.3)	4.1 (1.9)	5.6 (2.4)
Special needs designation, *n*(%)	23 (2.6)	6 (0.8)	17 (12.9)
Early Development Instrument (EDI)			
*Physical health and well-being, mean (SD)*	9.1 (1.1)	9.4 (1.2)	8.8 (1.4)
*Social competence, mean (SD)*	8.8 (0.6)	9.0 (1.6)	8.6 (1.8)
*Emotional maturity, mean (SD)*	8.5 (1.3)	8.8 (1.5)	8.3 (1.5)
*Language and cognitive development, mean (SD)*	8.8 (0.8)	9.0 (1.5)	8.5 (1.9)
*Communication skills and general knowledge, mean (SD)*	9.1 (1.7)	9.2 (2.1)	8.9 (2.3)

Non-linear models were fitted using splines (*p* = 0.03). Total daily screen use was associated with overall vulnerability in school readiness as measured by the EDI (unadjusted OR = 1.52, 95% CI: 1.12 to 1.89, *p* < 0.001; adjusted OR = 1.14, 95% CI: 1.01 to 1.54; *p* = 0.05; Table 2). It was estimated that the odds of being classified as vulnerable on the EDI are increased by 14% for every additional hour of screen use, after adjusting for the other variables in the model.

**Table 2:** Logistic regression analysis between total daily screen use and overall vulnerability (*n* = 876), as measured by the EDI

Exposure	Adjusted ^a^	
	**OR (95% CI)**	***p*** **-value**
Total daily screen use (hours)	1.14 (1.01 to 1.54)	**0.05**
**Covariates**		
Sex	1.58 (1.02 to 2.04)	**0.01**
Age (at outcome)	0.92 (0.51–1.67)	0.79
Physical activity	0.64 (0.30–1.35)	0.24
Maternal education	1.16 (0.73–1.84)	0.53
Maternal ethnicity	0.88 (0.63–1.22)	0.44
Special needs status	0.80 (0.65–0.99)	0.06
Annual household income	0.96 (0.69–1.33)	0.80

*Notes*. ^a^Model adjusted for child age (at outcome), sex, physical activity, maternal education, maternal ethnicity, follow-up period, special needs status (a previous developmental diagnosis identified using special needs designation reported by teachers on the EDI), and annual household income

For the secondary analyses, increased daily hours of screen use was associated with reduced domain scores for language and cognitive development (Table 3; β= -0.73, 95% CI = -1.13 to -0.23, *p* = 0.004). There was no evidence of an association between total daily screen use and the other EDI domains (*p* > 0.05). Adverse associations emerged between high screen users (top 10%) and language and cognitive development domain (β = -0.227, 95% CI = -0.37 to – 0.07; *p* = 0.004), and the communication skills and general knowledge domain (β= -0.03, 95% CI = -0.06 to -0.001; *p* = 0.042; Table 4). There was no evidence of interaction effects by age or sex (*p* > 0.05) in any of the models (Supplementary Table 1). There were 139 participants whose EDI outcome data were collected during the COVID-19 pandemic; we assessed whether results meaningfully differed using the data collected pre- and during the COVID-19 pandemic using a binary variable. Results from the sensitivity analyses (*p* = 0.67) suggested the data from these children be included..

**Table 3:** Linear regression analysis between total daily screen use and school readiness among children in kindergarten (*n* = 876)

Exposure variable	Physical Health and Well-Being		Social Competence		Emotional Maturity		Language and Cognitive Development		Communication Skills and General Knowledge	
	*β (p-*value) *95% CI*		*β (p*-value) *95% CI*		*β (p*-value) *95% CI*		*β (p*-value) *95% CI*		*β (p*-value) *95% CI*	
	*Unadj*	*Adj* ^a^	*Unadj*	*Adj* ^a^	*Unadj*	*Adj* ^a^	*Unadj*	*Adj* ^a^	*Unadj*	*Adj* ^a^
Total daily screen use (hours)	-0.02 (0.05) -0.12 to 0.04	-0.14 (0.08) -0.29 to 0.03	-0.09 (0.16) -0.21 to 0.06	-0.13 (0.78) -0.56 to 1.03	-0.09 (0.18) -0.73 to 1.21	-0.18 (0.06) -0.93 to 1.33	-0.52 (**0.002**) -1.00 to -0.03	-0.73 (**0.004**) -1.13 to -0.23	-0.12 (**0.04**) -0.20 to -0.004	-0.14 (0.09) -0.27 to 0.04

*Notes*. ^a^Model adjusted for child age (at outcome), sex, physical activity, maternal education, maternal ethnicity, follow-up period, special needs status (a previous developmental diagnosis identified using special needs designation reported by teachers on the EDI), and annual household income

**Table 4:** Linear regression analysis between screen use and school readiness among high screen users in children in kindergarten (*n* = 85)

Exposure variable	Physical Health and Well-Being		Social Competence		Emotional Maturity		Language and Cognitive Development		Communication Skills and General Knowledge	
	*β (p*-value) *95% CI*		*β (p*-value) *95% CI*		*β (p*-value) *95% CI*		*β (p*-value) *95% CI*		*β (p*-value) *95% CI*	
	*Unadj*	*Adj* ^a^	*Unadj*	*Adj* ^a^	*Unadj*	*Adj* ^a^	*Unadj*	*Adj*^a^	*Unadj*	*Adj*^a^
Top 10% of screen users b	-0.05 (0.28) -0.13 to 0.04	-0.03 (0.54) -0.16 to 0.06	-0.33 (0.16) -0.75 to 0.14	-0.22 (0.25) -0.63 to 0.22	-0.09 (0.17) -0.21 to 0.07	-0.07 (0.26) -0.19 to 0.06	-0.69 (**0.02**) -1.12 to -0.30	-0.23 (**0.004**) -0.37 to -0.07	-0.01 (0.03) -0.03 to -0.01	-0.03 (**0.04**) -0.06 to -0.001

*Notes*. ^a^Model adjusted for child age (at outcome), sex, ethnicity, physical activity, maternal education, special needs status (previous developmental diagnosis, follow-up period, special needs designation), and annual household income. ^b^Daily screen use = 8+ hours (*n* = 85)

## Discussion

Healthy development in early childhood helps set the state for positive emotional, social, and physical well-being. Children who are vulnerable in the early years are more likely to have poor future educational outcomes and are at increased risk for health issues such as obesity, heart disease and poor mental health [48, 49]. In our sample, it was also determined that for every 1-hour increase in screen use, the odds of being classified as vulnerable increased by 14%. Also of note is that daily total mean screen use among participants was 4.8 h, which is consistent with national data reported by the Health Behaviour in School-Aged Children Study which reported children spending 4.6 h per day in total screen-based pursuits [50].

Higher levels of screen use were associated (note the small effect size) with poorer scores in language and cognitive development, and communication skills and general knowledge. This finding is important as it highlights an adverse association between young children’s screen use and language skills and cognitive outcomes, two key attributes required for early academic success. Mechanisms to explain this finding may be reduced opportunities for parent-child interaction and play prior to school, which is critical for early language development [51]. Findings from Madigan et al. [5] systematic review and meta-analysis (*n* = 42 observational studies, *n* = 18,905 participants, < 12 years) revealed that higher levels of screen use were negatively associated with child language (*r* = −0.14; 95% CI, −0.18 to −0.10), supporting the findings of our study. Ribner et al. [52] also founds that television viewing was negatively associated with children’s school readiness skills, and this association increased as family income decreased. Interestingly, work by Linebarger and Vaala [53] found that the presence of a competent co-viewer may in fact boost very young children’s language learning from screen-viewing, much like the ways these processes facilitate learning in live scenarios.

Findings elicited from the examination between screen use and EDI outcomes among high screen users were in the anticipated direction, with increased screen use being associated with decreased language, communication, and cognitive domain scores. A review by Poitras et al. [2] (*n* = 96 studies) reported that increased screen use in children was weakly associated with delayed cognitive development and psychosocial health. Though examined among a slightly older age group (8-11 years), Walsh and colleagues reported that participants (*n* = 4,520) who met national screen use guidelines of ≤2 h/day displayed superior global cognition compared to those who did not [54], highlighting the importance of limiting excessive daily screen use in school aged children. Contrasting findings, however, were reported in a systematic review and meta-analysis (*n*= 58 studies) by Adelantado-Renau et al., which noted that the amount of time spent on overall screen media use was not associated with academic performance (ES = -0.29; 95% CI, -0.65 to 0.08), though the age span investigated was wide (4-18 years) [55]. The evidence to date is mixed, and while the previously listed studies do not provide evidence that could address the mechanism of the association between the screen use and poor language and cognitive outcome, they collectively suggest that when young children are observing screens, they may be missing important opportunities to master communication, interpersonal, and physical skills leading to developmental delays. For instance, when children use screens without an interactive or physical component, children are typically sedentary when they use screens potentially displacing opportunities to refine gross motor skills. Key to fostering optimal development in early childhood are the interactions between young children and their parents/guardians, teachers, or peers [48, 49]; unfortunately, screens have the propensity to limit such communication opportunities [50]. Consequently, it is possible that the outcomes in our study are the result of very similar pathways.

### Strengths & Limitations

A strength of this study is the large sample size of young children followed prospectively, teacher-reported validated outcome measures of school readiness with cut points for vulnerability, and continuous domain scores. The methodological approaches utilized to account for non-normal residuals and missing data also served as an important strength of this paper. The chief limitation of this study was the use of parent-reported screen use data which may be subject to recall bias and an underestimation of children’s screen time [56]. As well, approximately 17% of the SK sample was classified as vulnerable per the EDI tool, which is lower than current provincial estimates of 29.4%, and thus potentially limiting the generalizability of our results [57]. Given the differences between some of the adjusted and unadjusted estimates, residual confounding should be highlighted as a possible limitation. Future studies may consider addressing mediating factors like sleep and weight status. The generalizability of these findings is limited by the fact that the majority of participants identified as having parents that were highly educated, earned higher incomes and were of European descent. As well, we relied on a single-use measure to assess children’s screen use, absent of collecting nuanced information regarding content or context.

### Future Directions

Garnering more detailed data on the link between screen type or platform, screen content, and screen use duration may widen our comprehension of how screen use in the early years impacts school readiness, particularly given existing reports that support tentative positive outcomes of screen use among this young cohort (e.g., learning skills, face-to-face communication with educators and relatives, etc.). Likewise, it is also possible that some negative outcomes may also be driven by harmful screen content. In the context of COVID-19, there is a growing reliance on screen-based technology (e-learning, socialization, babysitting, etc.); consequently, finding novels ways to support screen use among young children to maximize the benefits and minimize the harms is particularly important [58]. While not a specific research objective in our study, the finding that there was no difference in the relationship between children’s screen use and EDI outcomes between 2020 and previous years is one that deserves further pursuit in the context of increased screen use through online learning (i.e., “virtual learning” or “e-learning”). Though some associations were found to be statistically significant in this study, they may not be clinically relevant; caution should be taken when interpreting the findings of this work given the small effect sizes reported. Lastly, to further expand the applicability of this work, future studies may consider running similar work with non-English speaking families to ascertain differences in impact of screen use on school readiness.

## Conclusions

Children with higher screen time may be at risk of vulnerability in teacher reported developmental readiness for school in kindergarten, particularly with language and cognitive development. Stronger associations were seen among children who were high daily users of screens. Next steps include understanding the context, type, and quality of screen use and school readiness outcomes, to develop and evaluate focused interventions to maximize the benefits and minimize the harms associated with screen use in early childhood.

## Supplementary Information

**Additional file 1.**

## Data Availability

The datasets used during the current study are available from the corresponding author on reasonable request.

## Abbreviations

EDI: early development instrument
JK: junior kindergarten
SK: senior kindergarten
