# Supplementary material for: Children’s screen use and school readiness at 4-6 years: prospective cohort study
Source: BMC Public Health. 2022 Feb 23;22:382. doi: 10.1186/s12889-022-12629-8 (PMC8864975; doi:10.1186/s12889-022-12629-8)
Supplement: Supplementary file 1 — Additional file 1. [file 12889_2022_12629_MOESM1_ESM.docx]

**Supplemental Table 1:** *P*-values for interaction effects by exposure (total daily screen use), in adjusted models for the continuous EDI domain scores.

|  | **EDI outcome** | | | | |
| --- | --- | --- | --- | --- | --- |
|  | **Physical health and well-being** | **Social competence** | **Emotional maturity** | **Language and cognitive development** | **Communication skills and general knowledge** |
| Global interaction likelihood ratio test | 0.65 | 0.24 | 0.31 | 0.21 | 0.25 |
| Age | 0.65 | 0.11 | 0.20 | 0.09 | 0.13 |
| Sex | 0.58 | 0.54 | 0.92 | 0.96 | 0.22 |
